# Robust discovery of periodically expressed genes using the laplace periodogram

**DOI:** 10.1186/1471-2105-10-15

**Published:** 2009-01-11

**Authors:** Kuo-ching Liang, Xiaodong Wang, Ta-Hsin Li

**Affiliations:** 1 Department of Electrical Engineering, Columbia University, New York, NY 10027, USA; 2 IBM T J Watson Research Center, Department of Mathematical Sciences, Yorktown Heights, NY 10598, USA

## Abstract

**Background:**

Time-course gene expression analysis has become important in recent developments due to the increasingly available experimental data. The detection of genes that are periodically expressed is an important step which allows us to study the regulatory mechanisms associated with the cell cycle.

**Results:**

In this work, we present the Laplace periodogram which employs the least absolute deviation criterion to provide a more robust detection of periodic gene expression in the presence of outliers. The Laplace periodogram is shown to perform comparably to existing methods for the *Sacharomyces cerevisiae* and *Arabidopsis* time-course datasets, and to outperform existing methods when outliers are present.

**Conclusion:**

Time-course gene expression data are often noisy due to the limitations of current technology, and may include outliers. These artifacts corrupt the available data and make the detection of periodicity difficult in many cases. The Laplace periodogram is shown to perform well for both data with and without the presence of outliers, and also for data that are non-uniformly sampled.

## Background

In the past decade, time-course gene expression datasets have become increasingly available, and have enabled the study of the dynamical behaviors of gene expression and the related regulatory mechanisms, as well as the analysis of the relationships between genes and cellular processes. Of particular interests are the genes that regulate and that are being regulated in relation to the cell-division cycles. A cell-division cycle is a series of sequential steps which are repeated throughout the lifetime of an eukaryotic cell, and it consists of four distinct phases: G_1 _phase, S phase, G_2 _phase, and M phase. The cell-division cycle is regulated by a complex interaction of a set of mechanisms which include genes such as cyclins and cyclin-dependent kinases (CDKs). These genes are known to be expressed periodically with respect to the cell-division cycle [1]. In the genome-wide time-course gene expression studies performed on *Saccharomyces cerevisiae *by [2,3], a group of genes are shown to be periodically expressed with respect to the cell-division cycle. Since then, other time-course gene expression studies have been conducted on other organisms such as *Plasmodium falciparum *[4] and *Schizosaccharomyces pombe *[5], and similar experiments were also conducted on human cells [6,7]. The detection of these periodically expressed genes is essential in the understanding of the regulatory mechanism for the cell-division cycle.

In recent years, many periodic signal detection algorithms have been proposed to detect periodically expressed genes from their time-course gene expression data. It is well known that for uniformly-spaced samples, the classical periodogram can be used to estimate the angular frequency spectrum of the sampled signal. Given a time sequence *y*_1_, ..., *y*_*N*_, the classical periodogram is computed as

(1) $$G_{N}(\omega )=\frac{1}{N}{\left|\sum_{t=1}^{N}y_{t}\exp(-jt\omega )\right|}^{2}.$$

where *j *= $\sqrt{-1}$, and *ω *is the angular frequency. Such classical periodogram has been used in various studies to detect periodicity in experimental data [3,8-11]. Other algorithms have also been proposed for the detection of sinusoids in expression data. In [12], a single pulse model is proposed to model the periodic behavior in the time-series gene expression of *Saccharomyces cerevisiae*. In [13], partial least-squares regression is used to fit the data to a linear combination of sinusoids of various frequencies. A gene is then represented as a point in a multidimensional space whose coordinates are given by its coefficients for the cosine and sine functions. The strength of periodicity of the expression of a gene is determined by the distance of the point from the origin. In [14], periodic time-course gene expression are modeled by *Y*_*i *_= *μ*_*i *_+ *β*_*i *_*f*(*t*_*i *_- *τ*_*i*_) + *ε*_*i*_, where *f*(*t*) is a periodic function which is modeled by a linear combination of cubic B-spline basis. This approach allows the modeling of periodic gene expression that take forms other than sinusoids. In addition to periodicity detection algorithms, various clustering algorithms have been proposed to obtain subsets of genes that exhibit periodic behavior. These methods include support vector machine [15], singular value decomposition [16], and independent component analysis [17].

While the above listed algorithms have achieved varying degrees of success, they are often limited by factors such as being developed for a specific dataset, not being able to provide a ranking for genes, and the nature of the time-course gene expression. In particular, one problem that plagues the detection of periodic signals in time-course gene expression data is that the samples are typically non-uniformly spaced, which is caused by the cell arresting and measurement methods employed by the experiments. One approach to resolve non-uniform sampling is to extrapolate a continuous signal from the available samples, and obtain a set of uniformly-spaced samples of the data from the extrapolated signal. Various works have explored this option, such as linear interpolation [18], cubic interpolation on the log of the expression levels [19], and B-spline interpolation [20]. However, errors can be introduced during interpolation, which can lead to inaccuracies in ranking the periodicity of genes. To modify the classical periodogram so that it can treat observations obtained with non-uniform spacing, it was proposed in [21] to fit the data to sinusoidal signals with least-squares. The asymptotic distribution of the proposed periodogram is derived in [22]. In [23], it is shown that the Lomb-Scargle periodogram outperforms methods based on interpolation. The Lomb-Scargle periodogram is also used to detect periodicity or to estimate the angular frequency spectrum of non-uniformly spaced biological samples in [24,25], and it is used to detect genes exhibiting periodic time-course gene expression in [26].

While using the least-squares fitting to sinusoidal functions allows the treatment of non-uniformly spaced samples, it is also well known that the least-squares method is non-robust in the presence of heavy-tailed noise and outliers due to its assumption that noise is independently and identically Gaussian distributed [27]. It is shown in [28] that the performance of Lomb-Scargle periodogram degrades in the presence of heavy-tailed non-Gaussian noise. In [29], robust periodicity detection algorithms using three different estimators – the M-estimator with Tukey's biweight function, the least trimmed squares, and the minimum covariance determinant estimator – are proposed to detect periodic genes in the mussel species *Mytilus californianus*. These robust regression methods are shown to outperform the Lomb-Scargle periodogram in the presence of outliers.

In this paper, we propose the use of the Laplace periodogram [30] as an alternative to the Lomb-Scargle periodogram for the detection of periodic genes from time-course gene expression data. The Laplace periodogram, which we describe in the Methods Section, employs the least absolute deviation (LAD) fit to sinusoidal functions instead of the least squares used in the Lomb-Scargle periodogram. As we will show in the following sections, the Laplace periodogram achieves better performance than the Fourier-based algorithm proposed in [31] and the M-estimator-based algorithm proposed in [29] when impulsive noise is present in the *Saccharomyces cerevisiae *datasets of [2,3].

## Results and discussion

### Periodic gene detection without outliers

In this section, we compare the periodicity detection performance of the Fourier-score-based algorithm [31], M-estimator [29], and the Laplace periodogram using two sets of real data. First, we use the *Saccharomyces cerevisiae *Alpha (6075 genes), CDC15 (5673 genes), and CDC28 (6214 genes) experiments from [2,3]. The time-course gene expression datasets of these three experiments are obtained from Cyclebase.org . The expression datasets obtained have been normalized to a common scale based on the percentage of the cell division cycle where the sampling occurs. Furthermore, the magnitudes of the gene expression have also been normalized to have a standard deviation of one [32]. Next, we compare the performance of the Fourier-score-based algorithm and the Laplace periodogram using the circadian oscillation dataset for auxin signaling in *Arabidopsis*[33].

#### *Saccharomyces cerevisiae *datasets

We use the same three sets of benchmarks described in [31]. Set B1 contains 113 genes identified to be periodically expressed by the small scale experiments of [3,13]. Set B2 contains genes identified in the Chromatin IP studies in [34], with 50 genes that are in common with the benchmark set B1 removed, leaving a total of 352 genes. Set B3 contains genes identified by MIPS [35] as "cell cycle and DNA processing" related genes. Genes that are also annotated as meiosis and genes that are in common with set B1 are removed, resulting in a total of 518 genes in this benchmark set. In B2 and B3, the genes selected based on their association with other genes that are associated with the cell cycle, or genes that play a role in the cell cycle. Thus, it is expected that most of the genes in B2 and B3 are not periodic, and should not be detected as periodic by the algorithms.

For each experiment, we ranked the time-course expression of the genes at the normalized cell-division-cycle frequency using the p-values of the scores computed by each of the three algorithms. Since we have very small number of samples, we estimated the p-values of the scores at the normalized cell-division-cycle frequency by a bootstrap method similar to [36], that is, for each gene, we fix the sampling times and permute the expression values. A total of 1000 permutations are generated, and for each permuted time series the score is computed. The p-value is simply the ratio of the permuted sequences that produced scores higher than from the original time-course expression. By permuting the expression values with respect to their sampling times, we aimed to destroy the periodicity that may exist within the time series. If periodicity exists within the time series, it is unlikely that a randomly permuted sequence will recover that periodicity, thus the magnitude of the spectrum at the tested period will be reduced and unlikely to achieve greater magnitude than the magnitude of the original time series. If on the other hand periodicity does not exist in the time series, then neither the original nor the permuted sequences will likely to have large magnitude at the test period, therefore, the possibility of the permuted sequence having higher magnitude than the original sequence is quite high. Thus the lower the p-value, meaning that only a small ratio of the permuted sequences resulted in higher magnitude than by the original sequence, the more likely that the gene is periodically expressed.

**Figure 1:**
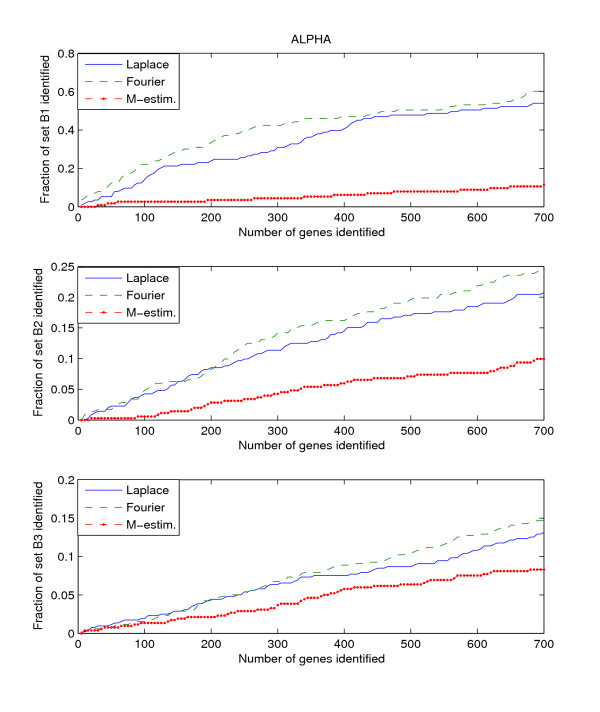
**Detection rate in the top scoring genes by the Fourier-score-based algorithm **[31]**, M-estimator **[29]**, and the Laplace periodogram for the Alpha dataset with no random impulse added for (a)B1, (b)B2, and (c)B3 benchmark sets**.

**Figure 2:**
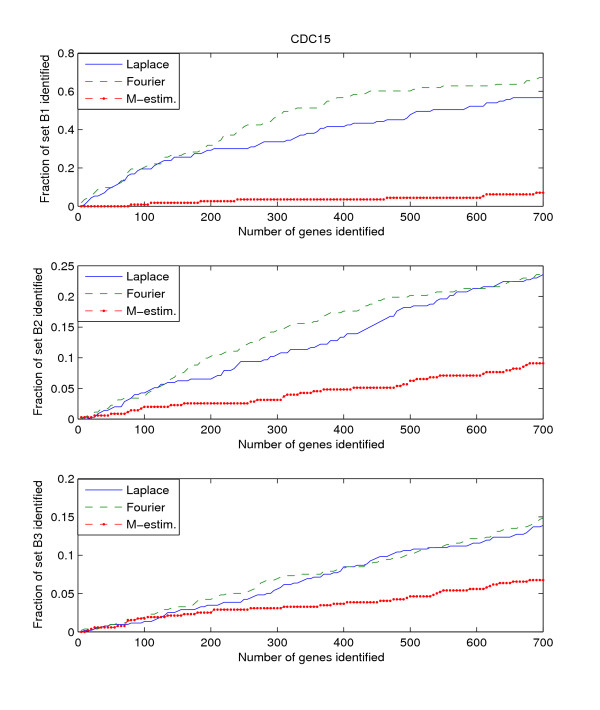
**Detection rate in the top scoring genes by the Fourier-score-based algorithm **[31]**, M-estimator **[29]**, and the Laplace periodogram for the CDC15 dataset with no random impulse added for (a)B1, (b)B2, and (c)B3 benchmark sets**.

**Figure 3:**
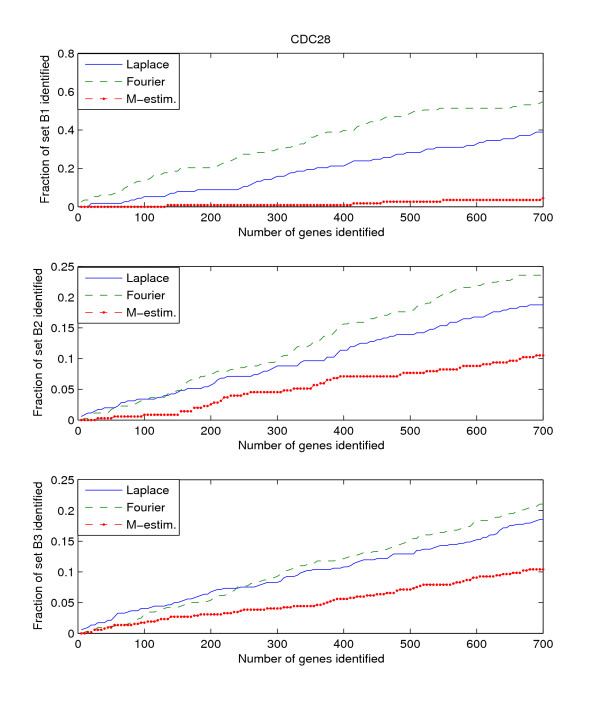
**Detection rate in the top scoring genes by the Fourier-score-based algorithm **[31]**, M-estimator **[29]**, and the Laplace periodogram for the CDC28 dataset with no random impulse added for (a)B1, (b)B2, and (c)B3 benchmark sets**.

Since the three benchmark sets discussed above include genes that are known to be or potentially periodically expressed, we will evaluate their performance by searching for the genes in these benchmark sets from amongst the highly ranked genes. We search within the top *K*-ranked genes for those genes that are present in the benchmark sets B1, B2, and B3. This search is performed for each of the three datasets, Alpha, CDC15, and CDC28. Thus we have a total of 9 "dataset – benchmark" combinations. For each combination, we plot the top *K*-ranked genes against the ratio of benchmark genes found in those top *K*-ranked genes, for *K *= 5 to *K *= 700. The plots for the 9 "dataset – benchmark" combinations are given in Figures 1, 2, 3.

The figures plot the ratio of periodic genes as indicated by the B1, B2, and B3 benchmark sets discovered in a subset of the top scoring genes scored by the three algorithms. As the subset of top scoring genes (number of genes in the subset) increases, the ratio of benchmark periodic genes contained in these subsets also increases.

From Figures 1, 2, 3, we can see that the Fourier score method consistently produces the best performance out of the three methods. The Laplace periodogram is able to detect periodically expressed genes at detection ratio that is comparable to the Fourier score for some experiment-benchmark combinations, and less for others. In particular, the Laplace periodogram has the biggest drop-off in performance compared to the Fourier score for the combinations of CDC15-B1 and CDC28-B1. For the other combinations, the Laplace periodogram either has comparable detection performance for all values of *K*, or is able to bridge the performance gap as *K *approaches 700. For each of the combinations, the M-estimator-based method achieves the worst performance, with large drop-offs in performance from both the Fourier score and the Laplace periodogram. Also note that the results in Figures 2 and 3 meet with our expectation that most of the genes in B2 and B3 are not periodic, leading to a very low ratio of genes from those two sets being detected as periodic.

#### *Arabidopsis *dataset

For this comparison, we use the experimental data provided by [33], which includes the expression of 22810 genes in *Arabidopsis*, 1677 of which are determined to follow a circadian fluctuation in mRNA abundance. Total of 12 samples of the time-series gene expression is taken for each gene, which covers approximately two cycles of the circadian oscillation.

**Figure 4:**
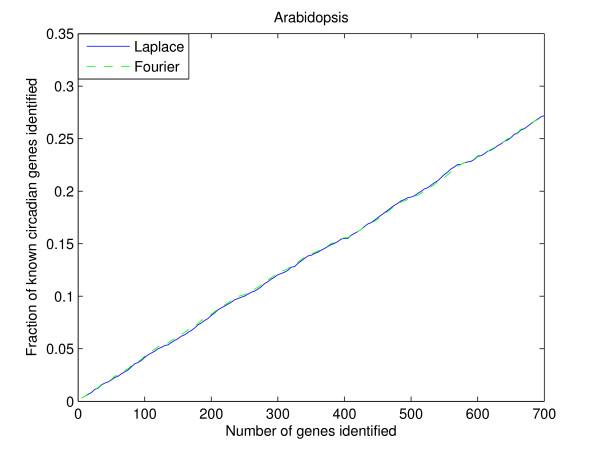
**Detection rate in the top scoring genes by the Fourier-score-based algorithm **[31]** and the Laplace periodogram for the *Arabdopsis *dataset**.

Comparison similar to the *Saccharomyces cerevisiae *datasets is also performed for the *Arabidopsis *dataset. A total of 250 permutations is computed at angular frequency of *π*. The prediction results for the Fourier-score-based algorithm and the Laplace periodogram are shown in Figure 4. As we can see from the figure, the performance of the two algorithms are virtually the same up to the 700 top scoring genes. For both algorithms, approximately 65% of the top 700 scoring genes are confirmed to be circadian according to experiments. Furthermore, the discovery ratio in the top 700 scoring genes for the *Arabidopsis *dataset is about 28%, whereas for the yeast Alpha-B1 "Dataset-benchmark" combination, the discovery ratio is about 82% in the top 700 scoring genes. It would seem at first easier to rank the gene expression correctly for *Arabidopsis *due to the higher ratio of genes in the dataset following circadian oscillation, at 7.35%, whereas for the Alpha-B1 "Dataset-benchmark" combination, the ratio of known periodic genes is only 1.86%. However, the *Arabidopsis *dataset also contains more genes than the yeast Alpha dataset, which increases the ranking difficulty. It should also be noted that the 28% of known circadian genes discovered in the top 700 genes is still a much higher detection ratio compared to one from a random ranking, which would on average discover about 3% in the first 700 genes.

### Periodic gene detection in the presence of outliers

**Figure 5:**
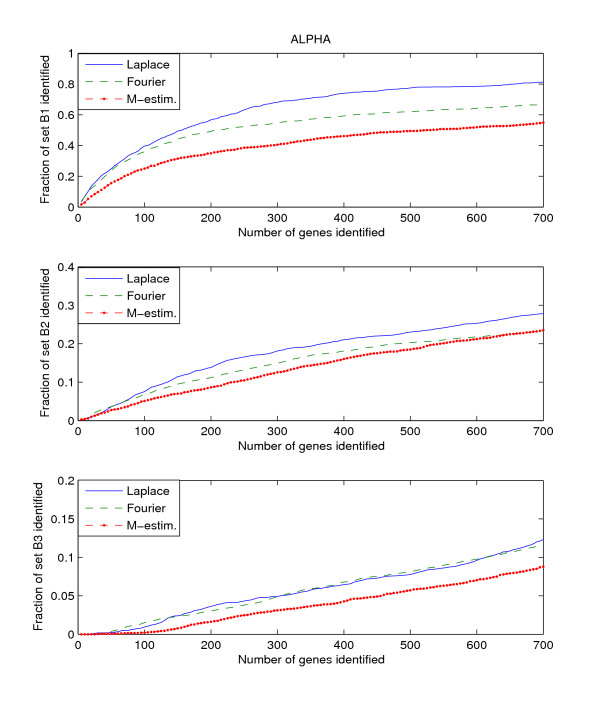
**Detection rate in the top scoring genes by the Fourier-score-based algorithm **[31]**, M-estimator **[29]**, and the Laplace periodogram for the Alpha dataset with random impulse added for (a)B1, (b)B2, and (c)B3 benchmark sets**.

**Figure 6:**
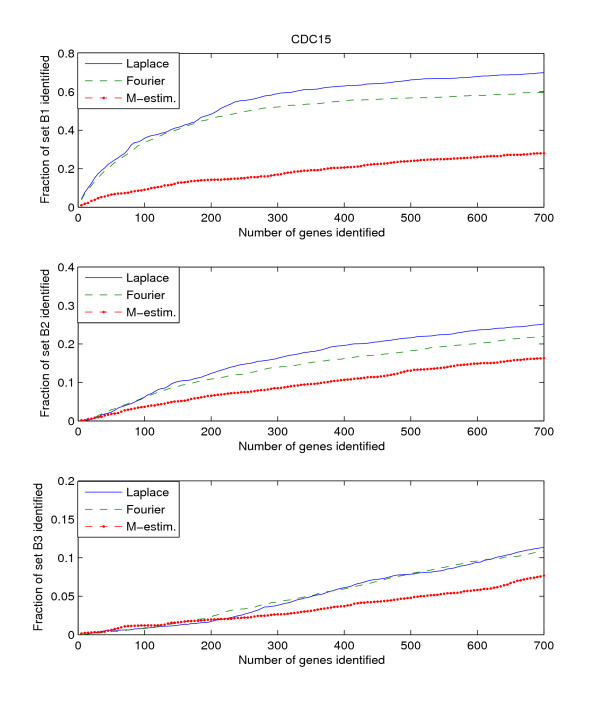
**Detection rate in the top scoring genes by the Fourier-score-based algorithm **[31]**, M-estimator **[29]**, and the Laplace periodogram for the CDC15 dataset with random impulse added for (a)B1, (b)B2, and (c)B3 benchmark sets**.

**Figure 7:**
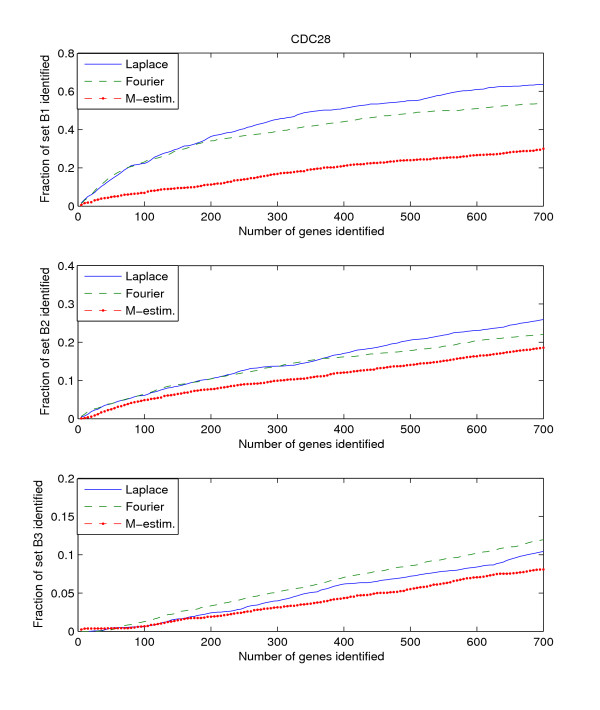
**Detection rate in the top scoring genes by the Fourier-score-based algorithm **[31]**, M-estimator **[29]**, and the Laplace periodogram for the CDC28 dataset with random impulse added for (a)B1, (b)B2, and (c)B3 benchmark sets**.

We now compare the detection performances of the Fourier score, Laplace periodogram, and M-estimator on the same *Saccharomyces cerevisiae *Alpha, CDC15, and CDC28 datasets from [2,3], but with added impulsive noise to the dataset. For each gene, there is a 0.1 chance of having an impulse noise at a random position of magnitude +7 or -7 in its time-course gene expression, replacing its original value. Note that the range of the peaks of the magnitude in these experiments varies between 3 and 5. We generate for each of the three experiments 50 such generated datasets with randomly placed impulse noises. We then perform periodicity detection with the three methods on these randomly generated datasets and averaged the results for each experiment. However, in our simulations, we observed that the performance for each of the algorithms when ranked using the estimated p-values is no better than if we had randomly ranked the genes. Therefore, instead of ranking the genes by their p-value, here we rank them by using their magnitude instead of using the estimated p-values. This observation can be explained by the presence of the outlier impulsive noise, whose influence on the spectrum is not removed by the random permutations, thus resulting magnitude for a periodically expressed time series and its permutations do not differ by a significant amount. The results of these plots are then given in Figures 5, 6, 7.

From these figures we can see that with the addition of impulse noise, the Laplace periodogram on average gives better detection performance than Fourier score for most of the combinations, and only in the CDC28-B3 combination does the Laplace periodogram achieves worse detection accuracy than Fourier score. However, it should be noted that a lot of the genes in benchmark set B3 are not involved in the transcriptional regulation, thus only a very small amount of genes in B3 are expected to be periodic [31]. What is surprising in these simulations is that even when impulse noises are added, the M-estimator-based method, which is designed to be robust in the presence of outliers, performs the worst out of the three methods compared. A closer look at the M-estimator proposed in [29], we see that the M-estimator is based on finding

(2) $$\tilde{\beta }=\underset{\beta }{\min}\sum_{t=1}^{N}\rho \left\{\frac{y_{t}-x_{i}^{T}\beta }{\sigma_{N}}\right\},$$

where *ρ *is a symmetric function with the Tukey's biweight function as its derivative, and *σ*_*N *_a scaling factor. The Tukey's biweight function is given as

(3) $$\psi (x)=\{\begin{array}{ll} x{\left(1-\frac{x^{2}}{c^{2}}\right)}^{2} & \text{for}|x|<c,\\ 0 & \text{for}|x|>c, \end{array}$$

where *c *is the constant that determines the shape of the curve and the cutoff point above which the residual of the sample and the current fit contributes zero weighting, thus completely removing the contribution of possible outliers that exceeds a certain magnitude. However, this approach requires a value for *c *which may vary from dataset to dataset. The optimal value for *c *may depend on the magnitude of the peaks and the magnitude of the outliers. When given an unknown dataset without a benchmark set of periodically expressed genes, it is uncertain how *c *can be tuned to achieve the optimal result, thus demonstrates a disadvantage of the M-estimator-based method. Note that for both simulations with and without the presence of outliers, we used the default value of *c *which came with the program provided by the authors of [29].

## Conclusion

Our simulation results have shown that the Laplace periodogram is a useful tool for detecting periodic time-course gene expression, particularly when the dataset contains outliers and when the sampling intervals are highly uneven. The Laplace periodogram achieves better performance for the *Saccharomyces cerevisiae *dataset when outliers are present, and achieves equal or comparable performances to the Fourier-based method for both the *Saccharomyces cerevisiae* and *Arabidopsis* datasets when no outlier.

## Methods

### Laplace periodogram

For time series samples ***y ***= [*y*_1_, ..., *y*_*N *_], the classical periodogram is computed as

(4) $$G_{N}(\omega )=\frac{1}{N}{\left|\sum_{t=1}^{N}y_{t}\exp(-jt\omega )\right|}^{2},$$

for the frequency range *ω *∈ (0, 2*π*). For frequencies $\omega =\frac{2\pi k}{N}$, *k *= 1, 2, ..., the classical periodogram can be alternatively written as

(5) $$G_{N}(\omega )=\frac{1}{4}N{\left\|{\tilde{\beta }}_{N}(\omega )\right\|}^{2},$$

where ${\tilde{\beta }}_{N}$ is given by

(6) $${\tilde{\beta }}_{N}≜\arg\underset{\beta \in {\mathbb{R}}^{2}}{\min}\sum_{t=1}^{N}{\left|y_{t}-x_{t}^{T}(\omega )\beta \right|}^{2},$$

With $x_{t}^{T}(\omega )≜[\cos(\omega t),\sin(\omega t)]$. In other words, ${\tilde{\beta }}_{N}$ is the solution to the least-squares regression of ***y ***to the regressor ***x***_*t*_[37].

To overcome the weakness of classical and Lomb-Scargle periodograms in dealing with outliers and heavy-tailed noise, it is proposed in [30] that the *L*_2 _norm in (6) be replaced with the *L*_1 _norm, thus replacing the least squares with least absolute deviation (LAD). Thus, the Laplace periodogram can be computed for $\omega =\frac{2\pi k}{N}$, *k *= 1, 2, ...,

(7) $$L_{N}(\omega )=\frac{1}{4}N{\left\|{\tilde{\gamma }}_{N}(\omega )\right\|}^{2},$$

where we replace the least squares coefficient ${\tilde{\beta }}_{N}$ with the LAD coefficient,

(8) $${\tilde{\gamma }}_{N}≜\arg\underset{\gamma \in {\mathbb{R}}^{2}}{\min}\sum_{t=1}^{N}\left|y_{t}-x_{t}^{T}(\omega )\gamma \right|.$$

Note here that the magnitude at each angular frequency can be computed independent of the other frequencies, meaning that if we know exactly the periodicity that we are looking for, there is no need to compute the LAD coefficients for the entire frequency spectrum. In [29], the proposed robust estimators are used in the Fisher's g-statistics, which has a denominator that is computed with contribution from all the frequency components. Thus the LAD periodogram has an advantage in terms of computational complexity when the periodicity is known, by foregoing the evaluation of the magnitudes of the rest of the frequency components.

An implementation of the proposed algorithm in MATLAB can be found at 

### Method for LAD approximation

To solve for the LAD coefficients, we can convert (8) into a set of equations and constraints to be solved using linear programming [38]. Let *γ *= [*γ*_1_*γ*_2_]^*T*^, and ***x***_*t *_= [*x*_*t*,1 _*x*_*t*,2_]^*T*^, where *x*_*t*,1 _= cos(*ωt*), and *x*_*t*,2 _= sin(*ωt*). We first consider the following equation,

(9) $$\begin{array}{cc} y_{t}-\sum_{j=1}^{2}\gamma_{j}x_{i,j}=u_{t}-v_{t}, & t=1,\cdots ,N, \end{array}$$

where *u*_*t *_and *v*_*t *_are non-negative variables. By setting *γ*_*j *_= *b*_*j *_- *c*_*j*_, where *b*_*j *_and *c*_*j *_are non-negative variables, we can obtain the best *L*_1 _approximation by solving the following linear programming problem:

(10) $$\begin{array}{ll} \text{Minimize} & \sum_{t=1}^{N}\left(u_{t}+v_{t}\right)\\ \text{subject to} & \begin{array}{cc} y_{t}=\sum_{j=1}^{2}(b_{j}-c_{j})x_{t,j}+u_{t}-v_{t}, & t=1,\cdots ,N, \end{array}\\ \text{and} & b_{j},c_{j},u_{t},v_{t}\geq 0. \end{array}$$

To solve the LAD approximation for non-uniformly spaced samples, we follow the same steps to solve for the LAD coefficient in the following,

(11) $${\tilde{\gamma }}_{N}≜\arg\underset{\gamma \in {\mathbb{R}}^{2}}{\min}\sum_{\tau =1}^{N}\left|y_{t_{r}}-x_{t_{r}}^{T}(\omega )\gamma \right|.$$

where [*t*_1_, *t*_2_, ..., *t*_*N *_] are the *N *non-uniformly sampled time instants.

In this formulation, the LAD coefficients can be easily solved using standard algorithms for solving linear programming problems. For our implementation in MATLAB, we used the LINPROG function in the Optimization Toolbox. In terms of computational time required to process the data, for experiment Alpha which consists of 6075 genes and 18 samples each, the total time to compute 1000 permutations for the p-value analysis takes approximately 24 hours on a Pentium Core 2 CPU at 2.66 GHz, which is similar to the amount of time taken by the M-estimator-based method, also implemented in MATLAB using the ROBUSTFIT function in the Statistics Toolbox.

## Authors' contributions

KL implemented the Laplace periodogram in MATLAB, performed the simulations and comparisions, and contributed in the writing of the draft. TL developed the Laplace periodogram in his earlier work. Both XW and TL conceived of the project and coordinated its implementation.
